# Family and Patient Psychoeducation for Severe Mental Disorder in Iran: A Review

**Published:** 2019-01

**Authors:** Yasaman Mottaghipour, Maryam Tabatabaee

**Affiliations:** 1 Department of Psychiatry, Shahid Beheshti University of Medical Sciences, Tehran, Iran.; 2 Department of Psychiatry, Tehran University of Medical Sciences, Tehran, Iran.

**Keywords:** *Developing Countries*, *Family Education*, *Low and Middle-Income Countries*, *Patient Education*, *Severe Mental Disorder*

## Abstract

**Objective:** There are evidence-based practices in the field of family and patient psychoeducation for patients suffering from severe mental disorders. However, given the variation in resources and cultural contexts, implementation of these services, especially in low and middle-income countries is faced with challenges.

This study aimed to review articles on family and patient psychoeducation of severe mental disorders in Iran and to find the characteristics of the main components necessary for the implementation of such practices in clinical settings.

**Method** **:** All published studies on family and patient psychoeducation for severe mental disorders (schizophrenia, schizoaffective, and bipolar disorder) conducted in Iran were searched up to May 2018; and key features and findings of each study were extracted and presented.

**Results: **Forty-eight studies were included in this review, of which 27 were randomized controlled trials, and 20 were quasi-experimental. One study was an implementation and service development report. The main findings of these studies were a significant decrease in relapse rate and/or rehospitalization rate and a significant decrease of burden and distress of families.

**Conclusion: **Despite a wide diversity in approaches, this review showed that different psychosocial interventions in which psychoeducation is one of their core and main components have promising results, demonstrating the significance of this intervention in Iranian mental health research. In our opinion, based on evidence, even with limited resources, it is no longer acceptable to deprioritize some forms of psychoeducation for patients and their families in clinical settings.

There are evidence-based practices in the field of family and patient psychoeducation for patients suffering from severe mental disorder (SMD). Clinical trials and systematic reviews have demonstrated that psychoeducation significantly reduces relapse and rehospitalization rates in patients with SMD as well as burden and stress level of caregivers (1, 2, 3). However, family and patient psychoeducation are not widely implemented in routine clinical practices, even in developed countries (4).

The main issues to be considered in the implementation of psychoeducation in routine clinical practices are staff skills, training, and follow-up supervision, applicability of the intervention to the service users, economic costs, and mental health team’s values and preferences (5, 6). Furthermore, implementation of these services, especially in low and middle-income countries (LMICs), is faced with challenges, given the variation in resources and cultural contexts. Education of participants, follow-up, and acceptability of services are few examples of barriers to feasibility that are mentioned in different articles (7).

Limited qualitative studies conducted in this area in Iran revealed that families of patients struggle with the lack of information on illnesses and how to deal with different issues related to them, while stigma is still a major concern for them (8, 9). In an overview of the first episode psychosis research in Iran, few studies related to aftercare services and psychosocial interventions showed promising results in reduction of relapse rates, distress level of relatives, and negative experience of caregivers(10).

Bipolar disorder occurs in 1% to 3.7% and schizophrenia in 1% of the general population (11, 12). The exact number of people suffering from SMD and their families is not available. There are about 60 million people in Iran from early adolescence to old age. Considering there are at least 4 people in a family, it is evident that a vast number of people are affected by SMD. 

For the past two decades, several studies have been conducted in the realm of patient and family psychoeducation in Iran. Finding information on different aspects of participants and programs, including level of education, type of intervention, and study design, can provide a framework for the implementation of such programs in routine clinical settings in LMICs.

This article aimed to review studies on family and patient psychoeducation of SMD in Iran and to find the characteristics of the main components necessary for the implementation of such practices in clinical settings. Demographic data of participants and different aspects of intervention used in psychoeducational research can highlight the need for future research and can also be used as a roadmap for mental health services.

## Materials and Methods

All published studies on family and patient psychoeducation for SMD (schizophrenia, schizoaffective, and bipolar disorder) conducted in Iran were searched up to May 2018. The electronic search was performed using PubMed, Scopus, Magiran, SID, PsychInfo, and Google Scholar. The following keywords in English and Farsi were used: psychoeducational family/patient intervention, family/patient psychoeducation, family/patient interventions, family/patient education and caregivers’ education/psychoeducation, combined with severe mental disorder/illness, schizophrenia, and schizoaffective bipolar disorder. The included papers were written in Farsi and English. Further, cross-reference searching for the purpose of obtaining more relevant studies was conducted. 

All studies on patient or family psychoeducation in SMD in Iran were included for this review. However, studies that developed a guideline were excluded. Both authors reviewed relevant studies and extracted data. Any disagreement was resolved by discussion. Where possible, authors of original papers were contacted for additional data. This review should not be considered as a systematic review, but rather as a review and description of key variables of studies supporting the implementation of psychoeducation for families and patients in routine clinical practice. 

Key features and findings of each study were extracted and presented in two tables. Data extracted on study characteristics included city of study, sample size, diagnosis, patients’ gender, relationship of family members to patient, family members’ education level, length and number of sessions, use of structured manual, attrition rate, type of intervention, personnel delivering intervention, study design, outcome measures, and main findings (family/patient). 

To collect information on different aspects of psychoeducational intervention from each paper, the following categorizations were employed to extract data on each variable:

- When different sections of a research were published in more than one paper, they were grouped together under 1 study with different dates/references.
- Sample size included the number of patients and/or family members participating in studies. The size of different arms of study were also reported if indicated in the paper.
- Patients’ gender was reported by percentage or number, the same as the original paper. Classification of the level of education in family members differed in studies and was presented by percentage or the majority of cases.
- Length of psychoeducational sessions showed the duration of psychoeducational intervention. Length of each session and number of psychoeducational sessions were also reported.
- Reporting of the use of structured manual variable was fitted into different categories. If the psychoeducational intervention was administered according to a manual, the manual reference was mentioned. If the content of the intervention was described based on each session, then “content of sessions described” was mentioned. Otherwise “information not given” was used. When pamphlets or other written materials were reported for psychoeducation, the phrase of “written information is given” was used.
- Given the wide diversity in reporting the attrition rate, it was stated the same as the original paper. Attrition rate included pre- and post-analysis dropout rates, response rate, and retention rate.
- With regards to type of intervention, all interventions other than “treatment as usual” (TAU) were listed. TAU usually comprised pharmacological treatment and inactive follow- up visits. Different interventions, including home visit/home care, social skills training, multiple family group” (MFG) psychoeducation, patient group psychoeducation, psychosocial rehabilitation, individual psychoeducation, telephone follow-up (TFU) and discharge planning, were reported.
- Whenever personnel delivering interventions based on different psychoeducational programs were mentioned in an article, they were included in this paper.
- Outcome measures used in each research as well as the main results of different interventions for patients and family members were reviewed. Main results were reported only where there were significant differences in outcome measures.

## Results

A total of 48 studies were included in this review. The results were presented in two tables. Table 1 demonstrates the details of the studies and interventions, sorted by study year and alphabetical order within any given year. Table 2 summarizes the outcome details. 

The study design in 27 of the studies was randomized controlled trial (RCT) and it was quasi-experimental in 20. One study was an implementation and service development report (76). The intervention used for the control group or the other arm of the study varied in different studies. It was treatment as usual in 31 studies (18-21, 25, 27-29, 34-37, 39-43, 45, 46, 49-51, 53-58, 61, 62, 65-67, 70-75, 78, 79), active intervention in four studies (22, 23, 26, 31, 32, 59, 60), and both treatment as usual and active intervention in five studies (38, 44, 47, 48, 64, 68). Seven studies had no control group (13, 15-17, 33, 69, 76), and one study had both control and placebo groups (14). The majority of participants’ entries was during hospitalization or after hospital discharge. 

The first study was published in 1995. Twenty-one studies took place in Tehran, 23 in the capital cities of provinces, two in towns (35, 69), and two were multicenter (64, 75). 

Sample sizes ranged from 15 to 270 in the included studies. Diagnosis of participants was SMD in 13 studies (13, 17, 27-29, 33, 40, 41, 47, 48, 55, 57, 61, 62, 64, 65, 68, 76), schizophrenia in 23 studies (14-16, 18-20, 31-33, 35-39, 42-46, 53, 54, 58-60, 66, 67, 71, 74, 78), and bipolar disorder in 11 studies (21, 25, 26, 34, 49, 50, 56, 69, 70, 74, 75, 79). First episode psychosis was included in 3 studies (22, 23, 33, 51). Two studies included child and adolescent patients (51, 70). Overall, 34 studies included a total of 3291 patients (43.9 SMD; 22.4% schizophrenia; 19.7% bipolar disorder, and 2% first episode psychosis).

Fourteen studies included no information on patients’ gender (16, 18-23, 25, 34, 35, 49, 50, 66, 69-71, 78, 79) From the 34 remaining studies, 27 included only male patients or the majority of patients were male (13-15, 17, 26-29, 31-33, 37-48, 55, 57, 58, 61, 62, 64, 65, 67, 68, 74, 76). The number of female patients was higher than or equal to male patients in seven studies (36, 51, 53, 54, 56, 59, 60, 72, 75). 

The participating family members were mostly parents, specifically mothers, followed by spouses and siblings. Family members’ education level is displayed in Table 1. Among studies that reported education level, only two included caregivers with no literacy (22, 23, 47 and 48). 

The length of each psychoeducation session varied from 45 minutes to four hours, but generally, it lasted between 90 to 120 minutes. The number of sessions varied from 1 to 14 sessions, and the maximum length of intervention was 12 months (17, 27-29, 49, 50, 64). However, the follow-up period of some studies extended up to three years (15). 

Eleven studies used a structured manual for psychoeducation (22, 23, 27-29, 33, 47, 48, 51, 57, 61, 62, 70, 72, 76, 79), 4 did not provide any information about the content of psychoeducational intervention (14, 17-20, 64), and the rest described the content of sessions. Four studies only provided written information for educational purposes (25, 31, 32, 64, 68). MFG psychoeducation was the most common psychoeducational intervention and was conducted in 31 studies (Table 1). In 16 studies, family psychoeducation was conducted during home visits. Furthermore, patients were present in all family psychoeducational sessions provided at home.

Five studies focused on patient psychoeducation and did not provide family psychoeducation (36, 42, 56, 72, 79). In the majority of the studies, psychoeducation was delivered along with other interventions, such as active follow- up, home visit, social skills training, crisis management, or psychosocial rehabilitation. Except for 1 study, in which family members were trained and worked as case managers (31, 32), other studies involved trained professionals for delivering psychoeducation. 

One study reported service development (76), therefore, had no outcome report in Table 2. Measures and scales that have been translated into Farsi and used in the studies are listed in Table 2. All studies found improvement in some outcome measures. In 16 studies, a significant decrease in relapse rate or rehospitalization rate was reported in the experimental group (13, 15, 18-21, 26, 31, 32, 44-46, 51, 55-57, 61, 62, 64, 68, 72). Further, 18 studies reported a significant decrease of burden and distress of families (22, 23, 25, 27-29, 31-33, 38-41, 47-50, 53, 54, 58, 65, 68, 69-71, 76).

## Discussion

This is the first review of patient and family psychoeducation for patients suffering from SMD in Iran. Despite wide diversity in approaches, this review shows that different psychosocial interventions, with psychoeducation as one of their core and main component, have promising results, demonstrating the significance of this intervention in Iran’s mental health research.

In 47% of the included studies, the diagnosis of patients was schizophrenia, however, the prevalence and number of beds in main psychiatric wards do not reflect the same statistics. Historically, family and patient psychoeducation first began with providing education to patients and families of patients suffering from schizophrenia. With a limited number of studies on patients suffering from bipolar disorder and first episode psychosis, there is a need to develop more specific psychoeducation interventions for these groups of families and their patients. 

The content of psychoeducation sessions in Iran was similar to programs in other parts of the world (2, 3, 11). Most articles mentioned adaptation from other references. Few papers detailed the content of the sessions based on each session or provided a structured manual reference. The point that needs to be considered is that the content of the information provided was brief due to the limited time of personnel and resources. 

MFG psychoeducation was presented without the presence of the patient. Cultural context plays a role in this format as families do not speak freely in front of the patients. Patients live with their families in Iran and families are the main caregivers, which is similar to other LMICs (80). With the exception of 6 studies (21, 42, 56, 72, 76, 79), which provided structured patient psychoeducation, other studies were conducted during home visits offered some form of education to family and their patient. When psychoeducation is delivered at home, program fidelity becomes a major issue. At home, there is less adherence to the protocol in terms of content and time spent for psychoeducation (63).

The prevalence of SMD is approximately the same for men and women. However, most participants in psychoeducation were male patients (27 studies out of 34 included studies that reported gender). Although the inpatient bed distribution is about 60% male to 40% female in psychiatric hospitals in Iran (personal communication with the Ministry of Health), research participants’ gender distribution still reflects a larger gap. Therefore, an investigation into the reasons why female patients’ participation rates are lower is important. For example, does stigma play a part in the gender participation rate (76)? Or, why do research samples include more male patients? On the other hand, the main caregivers were females, similar to other studies (3). For these reasons, looking into the involvement of male family members requires special attention. These considerations could increase participation rates in psychoeducation intervention, and hence provide better outcomes for patients and family members.

Studies were conducted at the capital cities of different provinces in Iran (12 provinces out of 31 provinces in Iran), and the majority were conducted in Tehran, the capital city of Iran. Some important questions are how many of the centers provide these services as a routine clinical practice? And how sustainable are psychoeducation programs? 

The specialty of those who delivered the services varied. In all studies, the intervention was delivered by professionals, except in 1 study in which family members delivered aftercare services, including psychoeducation (32). Keeping in mind that there are limited resources for family education, this seems to be another option for caregiving families, especially since it is also tested in different cultural settings (81).

Duration of psychoeducation in studies reviewed in this article ranged from 1 session to 14. In a number of studies in which aftercare/home visits were provided, the education provided to patients and families was mentioned. However, the format and duration of each session were not reported, which makes it difficult to reach any conclusions. To be able to continue to support and help patients and their families for a longer time, booster sessions and self-help groups are recommended within planning psychoeducation programs for families and patients in community settings. 

The list of outcome measures shows a number of questionnaires that were used in different studies, which have been translated and validated for use in Farsi. A set of the same questionnaires for patients as well as their families exist, which were administered in the studies and can be useful for future research in this area. The main significant results are listed in Table 2 for outcomes of family and patient psychoeducation. Although the design of most studies was quasi-experimental, with no randomization, results showed the same trend as other research conducted in these areas in other parts of the world (1, 2, 11).

Attrition rate is an important factor in planning the implementation of a program in clinical settings. Social and cultural issues can play a major role in the number of dropouts. Studies that were reviewed here reported attrition rates based on different definitions. Therefore, it is difficult to make a summary of the data. On the other hand, for each study that reported attrition rates, the number lied within an acceptable range compared to other research in this field. Research shows that culturally adapted interventions were more efficacious than the usual treatment in proportion to the degree of adaptation (82).

Psychoeducation is offered in different formats and packages in community settings. Given the mixed method and the use of other interventions beside the psychoeducation, which were employed by the majority of studies included in this paper, it is difficult to make a generalized inference of the results. Also, we cannot infer that the outcomes are attributed to psychoeducation per se. However, significant results are promising with regards to a number of important variables that were measured as outcomes for included studies. Some of these include a low rate of relapse and rehospitalization for patients (in 16 studies) as well as the decrease of the level of burden and distress of caregivers (in 18 studies). 

Another important issue to consider regarding the implementation of a psychoeducation program is the cost-effectiveness of such interventions. Three studies conducted in this area showed a lower cost in intervention groups (48, 64, 61). 

**Table 1:** Details of Studies on Family and Patient Psychoeducation

**Study, ** **Year**	**City of** **the study**	**Sample size** **(patient/family ** **member)**	**Diagnosis**	**Gender** **(patient)**	**Family member ** **relation/gender**	**Education** **(Family)**	**Length/ number of ** **psychoeducational ** **sessions**	**Use of structured ** **manual**	**Attrition rate**	**Intervention**	**Personnel**
Malakouti & Norouzi, 1995^13^ [in Farsi]	Zahedan	121 patients (94 schizophrenia, 6 schizoaffective 16 bipolar disorder, 3 acute psychosis, 2 other)	Severe mental disorder	83.5% male	Info not given	Info not given	Info not given	Content of sessions described	Info not given	Home visit	Psychologist & nurse
Khazaeili & Bolhari, 1996^14^ [in Farsi]	Tehran	30 family members (10 Exp†, 10 Ctrl‡, 10 Placebo)	Schizophrenia	Only male	Main caregivers	At Least 6 grades	6 sessions	Info not given	Info not given	MFG§	Psychologist
Malakouti et al. 1999^15^ [in Farsi]	Tehran	55 patients	Schizophrenia	70.9%male	Info not given	Info not given	3 sessions	Content of sessions described	Info not given	- MFG or individual psychoeduca tion & - Home visit & - TFU||	Psychiatry resident/ social worker/ psychologist
Assadollahi et al. 2000^16^	Isfahan	40 family members	Schizophrenia	Info not given	Only parents (20 fathers/ 20 mothers)	Majority primary school	Info not given	Content of sessions described	Info not given	MFG	Info not given
Sharifi et al. 2006^17^	Tehran	53 patients (19 schizophrenia, 34 bipolar disorder)	Schizophrenia & bipolar disorder	31 males	Parents and spouse	Info not given	Biweekly for 3 months then once a month for a year	Info not given	29 followed for 6 months or more	Home visit	GP¶ & social worker/ nurse
Fallahi, 2007^18^ [in Farsi] Fallahi & Kaldi, 2007^19^ ; ; Fallahi et al. 2009^20^ [in Farsi]	Tehran	48 patients (24 Exp, 24 Ctrl)	Schizophrenia	Info not given	Info not given	Info not given	6 sessions biweekly	Info not given	Info not given	Home visit	Nurse
Ghoreishiz adeh et al. 2008^21^	Tabriz	60 patients (30 Exp, 30 Ctrl)	Bipolar disorder	Info not given	Info not given	Info not given	6 sessions biweekly	Content of sessions described	Info not given	Individual patient and family psychoeduca tion	Info not given
Mottaghipo ur et al. 2008^22^; 2009^23^ [in Farsi]	Tehran	35 patients 62 family members (28 MFG /34 home visit)	First episode psychosis	Info not given	30.6% mother/21.0% father/ 25.8% sibling /11.3% spouse/ 3.2% children/ 8.1% others	39.4% no literacy or minimum	4 sessions	Mottaghipour, (2004)^24^	77% attended 4 sessions.	- MFG & TFU or - home visit	GP & social worker/ nurse
Dashtbozor gi et al. 2009^25^ [in Farsi]	Ahvaz	34 patients (17 Exp, 17 Ctrl)	Bipolar disorder, major depressive disorder	Info not given	Info not given	Info not given	6 sessions weekly	Written information and educational CD were given	3 Drop outs from control group	MFG	2 nurses
Fayyazi Bordbar et al. 2009^26^	Mashhad	60 patients (30 Exp, 30 home visit)	Bipolar disorder	78.2% male (79.3% in Exp group, 77.1% in Ctrl group)	Info not given	(43.4% below diploma/ 47.3% high school diploma/ others university degree) in Exp group	One session MFG then 4 Home visits every 3 months for follow- up	Content of sessions described	1 Drop outs from Exp group/ 2 from Ctrl group	-MFG & -home visit	Psychiatrist
Karmlou et al. 2009^27^; [in Farsi] 2010^28^; 2010^29^ [in Farsi]	Tehran	30 patients (15 Exp, 15 Ctrl) 31 family members	Severe mental disorder	61.3% male	18.7% mother/ 12.5% father/ 6.2% spouse/ 31.3% siblings/ 31.3% children	(37.5% primary school/ 18.8% secondary school/ 25% high school/ 18.7 university degree) in Ctrl group	6 sessions weekly	Mottaghipour, (2015)^30^	5 Drop outs from Exp group	MFG	2 psychologists
Malekouti et al. 2009^31^; 2009^32^ [In Farsi]	Tehran	129 patients (65 family members as case manager /64 professional case manager)	Schizophrenia	90 males	Info not given	Info not given	Once a month for 12 months	Written information is given	117 completed (73%).	-Family member home visit or -professional home visit	Community family member/ mental health worker
Mottaghipo ur et al. 2009^33^	Tehran	172 patients, 206 family members	Severe mental disorder/ first episode psychosis	61% male	32.8% mother	Info not given	1 session	Mottaghipour, (2004)^24^	34 Family members post-test after 6 months	MFG	Psychiatry resident/ psychiatrist & psychologist
Omranifard et al. 2009^34^ [in Farsi]	Isfahan	48 patients (24 Exp, 24 Ctrl)	Bipolar disorder	Info not given	Mainly spouses in Exp group/ mainly mothers in Ctrl group	At least literate	14 sessions, 4 weekly, and 10 biweekly	Content of sessions described	No dropouts	MFG	2 mental health workers
Shokraneh & Ahmadi, 2009^35^ [in Farsi]	Najafabad	30 patients (15 Exp, 15 Ctrl)	Schizophrenia	Info not given	Info not given	Info not given	6 sessions	Content of sessions described	No dropouts	MFG	Clinical Psychologist
Yasrebi et al. 2009^36^	Tehran	60 patients (30 Exp, 30 Ctrl)	Schizophrenia	Only female	Not applicable	Not applicable	Info not given	Content of sessions described	Info not given	Patient psychosocial rehabilitation	Info not given
Khankeh et al. 2010^37^ [in Farsi]	Hamedan	36 patients (18 Exp, 18 Ctrl)	Schizophrenia	21 males	Info not given	Info not given	6 sessions, twice a week in hospital, then 6 session biweekly home visit	Content of sessions described	1 Drop outs from Exp group	-MFG & - home visit	Info not given
Koolaee & Etemadi, 2010^38^	Tehran	62 family members (21 psychoeducation/ 21 behavioural family management/20 Ctrl)	Schizophrenia	72.8% male	Only mothers	25.4% primary school/ 32.8% secondary school/ 41.8% university degree	12 sessions weekly	Content of sessions described	3 Drop outs from behavior al family managem ent group/ 2 from psychoedu cation group/ 2 from ctrl group	-MFG or -behavioural family management	Info not given
Lotfi Kashani et al. 2010^39^ [in Farsi]	Tehran	22 family members (11 Exp, 11 Ctrl)	Schizophrenia	68.2% male	Parents	31.8% middle school/ 45.5% high school diploma/ 22.7% university degree	10 sessions biweekly	Content of sessions described	Info not given	MFG	Info not given
Navidian et al. 2010^40^ [in Farsi] Pahlavanz adeh et al. 2010^41^ [in Farsi]	Isfahan	50 schizophrenia patients, 50 bipolar patients (25 Exp, 25 Ctrl)	Schizophrenia & bipolar disorder	58% male	47% parents/ 22% spouse/ 20% sibling/ 11% children/	42% primary school/ 58% high school diploma or more	4 sessions weekly	Content of sessions described	No dropouts	MFG	Nurse
Jannesari et al. 2011^42^ [in Farsi]	Isfahan	76 patients (38 Exp, 38 Ctrl)	Schizophrenia	68.4% male	Not applicable	Not applicable	8 sessions, 4 biweekly, and 4 monthly	Content of sessions described	Info not given	Patient group psychoeduca tion	Psychiatry resident/ psychiatrist
Khankeh et al. 2011^43^	Tehran	60 patients (30 Exp, 30 Ctrl)	Schizophrenia	Only male	Info Not given	Info not given	1 session for family, 3 sessions for patient, then, home visit for 6 months	Content of sessions described	Info not given	Home visit	Nurse & psychologist
Niksalehi et al. 2011^44^	Bandar abbas	62 patients (21 home visit/ 21 telephone follow-up/ 20 Ctrl)	Schizophrenia	52.4% male home visit/ 23.80% male TFU	Info not given	At least literate	6 sessions biweekly	Content of sessions described	Info not given	Home visit	Nurse
Ranjbar et al. 2011^45^ [in Farsi] Khaleghpar ast, et al. 2014^46^	Tehran	46 patients (23 Exp, 23 Ctrl)	Schizophrenia	60.9% male	(26.4% father/ 52.6% mother/ 10.5% spouse/ 10.5% sibling) in Exp group (11.1% father/ 83.3% mother/ 5.6% children) in Ctrl group	Info not given	6 sessions in hospital then 6 biweekly home visits	Content of sessions described	No drop outs	-Individual family psychoeduca tion in discharge planning program & -home visit	Nurse
Sharifi et al. 2011^47^; Barfar et al. 2017^48^	Tehran	160 patients (80 Exp, 80 Ctrl) 118 family members from Exp group (49 MFG/ 69 home visits)	Severe mental disorder	45 males in Exp group	(40% mother/ 18% father/ 25% sister/ 14% brother/ 10% spouse/ 6%children/ 4.2% others) in Exp group	32.2% no literacy or minimum	6 sessions weekly	Mottaghipour, (2015)^30^	56.8% attended four sessions and more.	-MFG or -home visit	GP & social worker
Mojarrad Kahani et al. 2012^49^ [In Farsi]; Mojarrad Kahani & Soltanian, 2013^50^ [in Farsi]	Mashhad	15 family members (6 Exp, 9 Ctrl)	Bipolar disorder	Info not given	20% spouse/ 66% parents/ 14% sibling	20% primary school/ 40% middle school/ 26% high school diploma/ 14% university degree	12 sessions weekly	Content of sessions described	No drop outs	MFG	Info not given
Shahrivar et al. 2012^51^ [in Farsi]	Tehran	40 patients (adolescents) (20 Exp, 20 Ctrl)	First episode psychosis	38.9% male in Exp group, 35% male in Ctrl group	Mainly mothers	Info not given	4 sessions weekly	Mahmudi Gharaee, (2011)^52^	2 drop outs from Exp group	-MFG & -TFU	Info not given
Sharif et al. 2012^53^ ; Shaygan & Sharif, 2013^54^ [in Farsi]	Shiraz	70 patients (35 Exp, 35 Ctrl)	Schizophrenia	63% female Exp group, 43% female Ctrl group	Mainly mothers	Majority primary school	10 sessions twice a week	Content of sessions described	2 Drop outs from Exp group/ 3 from Ctrl group	MFG	Psychiatric nurse/ psychiatrist
Sharifi et al. 2012^55^	Tehran	130 patients (66 home care/ 64 Ctrl) (70 bipolar disorder/ 60 schizophrenia and schizoaffective)	Severe mental disorder	33.3% female in home care group, 32.8% female in Ctrl group	Info not given	Info not given	12 sessions monthly	Content of sessions described	77.4% remained in home care service for 12 months	Home visit	GP & social worker
Javadpour et al. 2013^56^	Shiraz	108 patients (54 Exp, 54 Ctrl)	Bipolar disorder	(22 male, 23 female) in Exp group, (20 male, 21 female) in Ctrl group	Not applicable	Not applicable	8 sessions weekly	Content of sessions described	86 Completed.	-Individual patient psychoeduca tion & -TFU	Psychiatry resident
Barekatian et al. 2014^57^	Isfahan	123 patients, (61 Exp, 62 Ctrl)	Severe mental disorder	40 males in exp group	Info not given	Info not given	6 sessions weekly	Mottaghipour, (2015)^30^	9 Drop outs from Exp group	- MFG & - home visit or TFU	GP & clinical psychologist
Fallahi et al. 2014^58^	Tehran	71 family members (36 Exp, 35 Ctrl)	Schizophrenia	86.1% male in Exp group, 82.9% male in Ctrl group	(11.1% spouse/ 83.4 parents /2.8% sibling / 2.8% children) in exp group	Info not given	4 sessions weekly	Content of sessions described	31 completed	MFG	Psychiatric nurse
Khirabadi et al. 2014^59^; Omranifard et al. 2014^60^	Isfahan	60 family members (30 Need-based psychoeducation/ 30 textbook content psychoeducation)	Schizophrenia	15 males in exp group, 19 males in ctrl group	Info not given	Info not given	10 sessions biweekly	Content of sessions described	20 Completed in Exp group; 22 Completed in Ctrl group	Need-based MFG or -textbook content psychoeduca tion	2 psychiatry residents in Exp group; 2 nurses in Ctrl group
Ghadiri Vasfi et al. 2015^61^; Moradi- Lakeh et al. 2017^62^	Tehran	120 patients (60 Exp, 60 Ctrl)	Severe mental disorder	37% female in exp group, 28% female in ctrl group	Info not given	68% high school or university degree Exp group; 47% high school or university degree in Ctrl group	6 sessions weekly	Mottaghipour, (2010)^63^	3 drop outs form Exp group	- MFG & - TFU or home visit & - SST# for Patients	Info not given
Malakouti et al. 2015^64^	Multicenter (Tehran & Karaj)	176 patients (66 GP as case manager, 57 nurses as case manager, 57 Ctrl)	Severe mental disorder	63% male in GP group, 55.7% male in nurse group, 55.5% male in Ctrl group	Info not given	Info not given	12 sessions monthly	Written information is given	20 lost to follow- up in GP group, 5 lost to follow- up in nurse group, 3 lost to follow- up in Ctrl group	-GP group home visit or -nurse group home visit	GP/ nurse
Mami et al. 2015^65^ [in Farsi]	Ilam	44 family members (22 Exp, 22 Ctrl)	Psychotic disorders	68.2% males	Only parents, 86.4% mothers	31.8% middle school, 45.5% high school diploma, 9.1% university degree	4 sessions biweekly	Content of sessions described	Info not given	MFG	Info not given
Rahmani et al. 2015^66^	Tabriz	74 family members (37 Exp, 37 Ctrl)	Schizophrenia	Info not given	37.5% parents	70.3% high school diploma	8 sessions, 3 times a week	Content of sessions described	2 drop outs form each group	MFG	Nurse
Vaghee et al. 2015^67^ [in Farsi]	Mashhad	60 patients (30 Exp, 30 Ctrl)	Schizophrenia	93.3% males in Exp group, 83.3% males in Ctrl group	(36.7% mother 30% father 13.3% sister 13.3% spouse 3.3% children 3.3% others) In Exp group	At least 9th grade (46.7% middle school/ 40% high school diploma/ 13.3% university degree) in Exp group	2 sessions in a week	Content of sessions described	2 Drop outs form each group before analysis	MFG	Nurse & psychologist
Malakouti et al. 2016^68^	Tehran	182 patients (60 home visit/ 61 TFU/ 61 Ctrl)	Severe mental disorder	60% male (56.7% male, home visit, 63.9% male TPU, 57.4% male in Ctrl group	Info not given	Info not given	12 sessions once a month in home visit group	Written information is given	16 Drop outs before analysis	- Home visit or - TFU	Nurse
Sazvar et al. 2016^69^ [in Farsi]	Kashan	40 family members	Bipolar disorder	Info not given	55% female	27.5% middle school, 50% high school, 22.5% university degree	10 sessions weekly	Content of sessions described	Info not given	MFG	Info not given
Sharif et al. 2016^70^	Shiraz	40 family members (adolescents) (20 Exp, 20 Ctrl)	Bipolar disorder	Info not given	38 mothers & 2 fathers	Info not given	6 sessions weekly	Mahmudi Gharaee, (2011)^52^	No drop out	MFG	Nurse and psychiatrist
Sheikholesl ami et al. 2016^71^ [in Farsi]	Rasht	30 family members (15 Exp, 15 Ctrl groups)	Schizophrenia	Info not given	58.3% female caregiver	79.2% high school	12 sessions, twice a week	Content of sessions described	3 Dropouts from each group	MFG	Psychologist
Faridhosse ini et al. 2017^72^	Mashhad	26 patients (13 in exp, 13 in Ctrl groups)	Bipolar disorder	6 males in exp group 7 males in ctrl group	Not applicable	Not applicable	8 sessions, twice a week	Tabatabaee et al, (2014)^73^	1 dropout from each group	Structured patient group psychoeduca tion	Info not given
Haji Aghaei et al. 2017^74^ [in Farsi]	Qazvin	100 family members (50 Exp, 50 Ctrl)	Schizophrenia	70 males	(23 males, 27 females) in each group	Info not given	8 sessions weekly	Content of sessions described	Info not given	MFG	Nurse
Pakpour et al. 2017^75^	Multicenter	270 patients (134 Exp, 136 Ctrl)	Bipolar disorder	44.8% male in Exp group 49.3% male in Ctrl group	Info not given	Info not given	2 sessions of MFG & 3 session of motivational interviewing over one month	Content of sessions described	9 lost to follow- up in Exp group and 7 in Ctrl group	-MFG & -motivational interviewing for patients	Psychiatrist and psychologist
Mirsepassi et al. 2018^76^	Tehran	77 patients	Severe mental disorder	75% male	Info not given	Info not given	6 family psychoeducation sessions weekly, 8 patient psychoeducation weekly	Tabatabaee et al, (2014)^73^; Mottaghipour et al, (2014)^77^; Mottaghipour, (2015)^30^	35% drop outs	-MFG & -structured patient group psychoeduca tion	Psychologists/ psychiatry resident & social worker/ nurse
Rezaei et al. 2018^78^	Tehran	100 family members (50 Exp, 50 Ctrl)	Schizophrenia	Info not given	Info not given	Info not given	10 sessions, twice a week	Content of sessions described	5 lost to follow- up in each group	MFG	Info not given
Saberi et al. 2018^79^ [in Farsi]	Rasht	30 patients (15 Exp, 15 Ctrl)	Bipolar disorder	Info not given	Not applicable	Not applicable	8 sessions weekly	Tabatabaee et al, (2014)^73^	3 dropouts from each group	Patient group psychoeduca tion	Info not given

† Exp = Experimental Group,

‡ Ctrl= Control Group,

§ MFG= Multiple Family Group,

|| TFU = Telephone Follow- up,

¶ GP= General Practitioner,

# SST= Social skills Training

**Table 2:** Outcome of Studies on Family and Patient Psychoeducation

**Study/ Year**	**Design**	**Outcome Measures**	**Main Results: Family**	**Main Results: Patient**
Malakouti & Norouzi, 1995^13^	Quasi-experimental	- Adherence to medication - Rehospitalization rate - Social and occupational function		- Increase in adherence to medication - Decrease in rehospitalization rate - Increase in social and occupational function
Khazaeili & Bolhari, 1996^14^	RCT†	Researchers-developed Questionnaires	- Increase in illness awareness - Decrease in negative attitude	Improvement in daily functioning
Malakouti et al. 1999^15^	Quasi-experimental	- Duration of hospitalization stay - Employment rate - Rehospitalization rate - Treatment cost		- Decrease in duration of stay - Increase in employment rate - Decrease in rehospitalization rate - Decrease in treatment cost
Assadollahi et al. 2000^16^	Quasi-experimental	Researcher-developed index: patient management skills	Improvement in patient management skills	
Sharifi et al. 2006^17^	RCT	Rehospitalization rate		86% not hospitalized.
Fallahi et al. 2007^18^; Fallahi & Kaldi 2007^19^; Fallahi et al. 2009^20^	Quasi-experimental	Rehospitalization rate		Decrease in rehospitalization rate
Ghoreishizadeh et al^21^. 2008	RCT	-Global Assessment of Functioning (GAF) - Rehospitalization rate -Relapse rate		- Decrease in rehospitalization rate - Decrease in relapse rate
Mottaghipour et al. 2008^22^^; ^2009^23^	RCT	-Client Satisfaction Questionnaire (CSQ-8) -Experience of Caregiving Inventory (ECI) -General Health Questionnaire (GHQ-28)	- Decrease in burden - Decrease in distress	
Dashtbozorgi et al. 2009^25^	RCT	-Bech-Rafaelsen Mania Scale -Compliance Rating Scale -Family Assessment Device -Global Assessment of Functioning (GAF) -Hamilton Depression Scale	Improvement in family assessment device score	
Fayyazi Bordbar et al. 2009^26^	RCT	-Duration of continuing medication -Number of follow up psychiatric visits -Relapse rate		- Increase in mean time of taking medications - Increase in follow up visits - Decrease in relapse rate
Karmlou et al. 2009^27^; 2010^28^; 2010^29^	Quasi-experimental	-Family Environment Scale (FES) -Family Questionnaire (FQ)	- Decrease in criticism - Increase in expressiveness and cohesion	
Malekouti et al. 2009^31^; 2009^32^	Quasi-experimental	-Family Experience Interview Schedule (FEIS) -General Health Questionnaire (GHQ) -Knowledge Questionnaire for Caregivers - Kohlman Evaluation of Living Skills (KELS) -Positive and Negative Symptom Scale (PANSS) -Rehospitalization rate -Wisconsin Quality of Life	- Decrease in burden in both groups after intervention - Increase in knowledge in both groups after intervention - Improvement in QOL in both groups after intervention	- 67% decrease in hospitalization rate compared to the year before in both groups. - Improvement in social skills in both groups - Decrease in psychopathology in both groups
Mottaghipour et al. 2009^33^	Quasi-experimental	- Experience of Caregiving Inventory (ECI) - General Health Questionnaire (GHQ-28)	- Decrease in burden - Decrease in distress	
Omranifard et al. 2009^34^	Quasi-experimental	- Family Burden Questionnaire -WHO Quality of Life (WHOQOL-BREF)	Improvement in quality of life	
Shokraneh & Ahmadi, 2009^35^	Quasi-experimental	Positive and Negative Symptom Scale (PANSS)		- Decrease in total, positive, negative, and aggression subscales score
Yasrebi et al. 2009^36^	Quasi-experimental	-Researcher-developed Social Skills Checklist -Scale for Assessment of Negative Symptoms (SANS)		- Improvement in social skills - Decrease in negative symptoms
Khankeh et al. 2010^37^	Quasi-experimental	-Heinrichs Quality of Life Scale (QLS) -Researchers-developed Self-control Checklist		Improvement in interpersonal dimension of QLS
Kolaee & Etemadi, 2010^38^	RCT	-Brief Psychiatric Rating Scale (BPRS) -Family Burden Interview Schedule (FBIS) -Family Questionnaire (FQ)	- Decrease in burden in psychoeducation group - Decrease in EE in behavioral management group	Decrease in positive symptoms in psychoeducation group
Lotfi Kashani et al. 2010^39^	Quasi-experimental	General Health Questionnaire (GHQ-28)	- Decrease in total score	
Navidian et al. 2010^40^ Pahlavanzadeh et al. 2010^41^	RCT	-Depression Anxiety Stress Scale (DASS) -Zarit Burden interview (ZBI)	- Decrease in DASS score - Decrease in family burden	
Jannesari et al. 2011^42^	RCT	-Global Assessment of Functioning (GAF) - Schizophrenia Quality of Life (SQLS)		- Improvement in GAF score - Improvement in QOLS score
Khankeh et al. 2011^43^	Quasi-experimental	Researchers-developed Self-care Checklist		Improvement in self- care
Niksalehi et al. 2011^44^	RCT	-Brief Psychiatric Rating Scale (BPRS) -Duration of hospitalization stay -Rehospitalization rate		- Improvement in BPRS - Decrease in length of stay - Decrease in rehospitalization rate
Ranjbar et al. 2011^45^ Khaleghparast et al. 2013^46^	RCT	-Discharge List (DL) -Knowledge Measurement Questionnaire (KMQ) -Rehospitalization rate	- Increase in knowledge level	- Improvement in clinical symptoms - Decrease in rehospitalization rate
Sharifi et al. 2011^47^; Barfar et al. 2017^48^	Quasi-experimental	-Client Satisfaction Questionnaire (CSQ-8) - Clinical Severity Index (CGI) - Experience of Caregiving Inventory (ECI) - General Health Questionnaire (GHQ-28) -Global Assessment of Functioning (GAF) -Hamilton Depression Rating Scale (HDRS) - Positive and Negative Symptom Scale (PANSS) -Rehospitalization rate -WHO Quality of Life (WHOQOL-BREF) -Young Mania Rating Scale (YMRS)	- Decrease in burden - Decrease in distress	Lower cost in intervention group
Mojarrad Kahani et al. 2012^49^; Mojarrad Kahani & Soltanian, 2013^50^	RCT	General Health Questionnaire (GHQ-28)	Improvement in total score	
Shahrivar et al. 2012^51^	RCT	- Children's Depression Inventory (CDI) - Children Global Assessment Scale (CGAS) -Global Assessment of Functioning (GAF) -Hamilton Depression Rating Scale (HDRS) - Kiddie-Schedule for Affective Disorders and Schizophrenia-Present and Lifetime (K-SADS- PL) - Positive and Negative Symptom Scale (PANSS) - Relapse rate - Rehospitalization rate -Young Mania Rating Scale (YMRS)		- Decrease in relapse rate
Sharif et al. 2012^53^ Shaygan & Sharif, 2013^54^	RCT	-Brief Psychiatric Rating Scale (BPRS) -Family Burden Questionnaire (FBIS)	Decrease in burden	Decrease in BPRS score
Sharifi et al. 2012^55^	RCT	-Client Satisfaction Questionnaire -8 (CSQ) -Global Assessment of Functioning (GAF) -Positive and Negative Symptom Scale (PANSS) -Rehospitalization Rate -WHO Quality of Life (WHOQOL-BREF) -Young Mania Rating Scale (YMRS)		- Increase in service satisfaction - Decrease in global illness severity - Decrease in psychotic symptoms - Decrease in rehospitalization rate
Javadpour et al. 2013^56^	RCT	-Bech Rafaelsen Mania Assessment Scale -Hamilton Depression Rating Scale (HDRS) -Medication Adherence Rating Scale -Rehospitalization rate -WHO Quality of Life (WHOQOL-BREF)		- Increase in medication compliance - Decrease in rehospitalization rate - Improvement in quality of life
Barekatian et al. 2014^57^	RCT	-Clinical Global Impression Severity Index (CGIS) -Global Assessment of Functioning (GAF) -Hamilton Depression Rating Scale (HDRS) -Positive and Negative Symptom Scale (PANSS) -Rehospitalization rate -WHO Quality of Life (WHOQOL-BREF) -Young mania rating scale (YMRS)		- Increase in GAF score - Decrease in HDRS Score - Decrease in rehospitalization rate
Fallahi et al. 2014^58^	RCT	Family Burden Questionnaire (FBIS)	Decrease in burden	
Khirabadi et al. 2014^59^; Omranifard et al. 2014^60^	Quasi-experimental	-Global Assessment of Functioning (GAF) Positive and Negative Syndrome Scale (PANSS) - Schizophrenia Quality of Life (SQLS) -WHO Quality of Life (WHOQOL-BREF)		- Improvement in GAF - Decrease in PANSS score - Improvement in psychosocial and symptom subscale of SQLS
Ghadiri Vasfi et al. 2015^61^ Moradi-Lakeh et al. 2017^62^	RCT	-Clinical Global Impression Severity Index (CGI) -Cost-effectiveness & cost-utility -Duration of hospitalization Stay -Global Assessment of Functioning (GAF) -Hamilton Depression Rating Scale (HDRS) -Positive and Negative Symptom Scale (PANSS) -Rehospitalization rate - WHO Quality of Life (WHOQOL-BREF) -Young Mania Rating Scale (YMRS)		- lower cost in intervention group - Decrease in hospitalization stay - Decrease in symptom severity (all indicators) - Decrease in rehospitalization rate
Malakouti et al. 2015^64^	RCT	- Client Questionnaire Satisfaction (CQS) - Cost Questionnaire - Family Experience Interview Schedule - General Health Questionnaire (GHQ-28) - Kohlman Evaluation of Living Skills (KELS) - Knowledge Questionnaire for Caregivers - Positive and Negative Symptom Scale (PANSS) -Rehospitalization rate - SF-36 Questionnaire -Young Mania Rating Scale (YMRS)	- Improvement in service satisfaction in both intervention groups - Improvement in caregivers’ knowledge in both intervention groups	- Higher cost in nurse group compared to GP and Ctrl group - Higher rehospitalization rate in Ctrl group - Improvement in young score in both intervention groups
Mami et al. 2015^65^	Quasi-experimental	General Health Questionnaire (GHQ-28)	- Improvement in anxiety, depression, and social dysfunction subscales	
Rahmani et al. 2015^66^	Quasi-experimental	Opinion about Mental Illness (OMI)	- Improvement in family attitude toward mental illness	
Vaghee et al. 2015^67^	RCT	Modified Version of Internalized Stigma of Mental Illness scale	- Decrease in stigma	
Malakouti et al. 2016^68^	RCT	-Client Questionnaire Satisfaction (CQS) -Family Experience Interview Schedule -General Health Questionnaire (GHQ-28) -Knowledge Questionnaire for Caregivers (FEIS) -Kohlan Evaluation of Living Skills (KELS) -Positive and Negative Syndrome Scale (PANSS) -Rehospitalization Rate -Short Form of Health Survey (SF-36) -Young Mania Rating Scale (YMRS)	- Decrease in burden - Increase in knowledge of schizophrenia and knowledge of bipolar	- Improvement in CSQ - Improvement in GHQ - Improvement in KELS - Improvement in PANSS - Decrease in rehospitalization rate
Sazver et al. 2016^69^	Quasi-experimental	The Level of Expressed Emotion Scale	- Decrease in expressed emotion	
Sharif et al. 2016^70^	RCT	- Mental Health Questionnaire - Quality of Life Questionnaire	- Improvement in mental health - Improvement in quality of life	
Sheikholeslami et al. 2016^71^	Quasi-experimental	-Family Assessment Device -Ryff Psychological Well-Being	- Improvement in family function and psychological well-being	
Faridhosseini et al. 2017^72^	RCT	- Compliance rate -Hamilton Depression Rating Scale(HDRS) -Rehospitalization rate -Relapse rate - Short Form Health Survey (SF36) -Young Mania Rating Scale (YMRS)		- Improvement in quality of life - Decrease in rehospitalization and relapse rates
Haji Aghaei et al. 2017^74^	RCT	Positive and Negative Symptom Scale (PANSS)		Decrease in PANSS score
Pakpour et al. 2017^75^	RCT	- Action and coping planning -Adverse Drug Reaction (ADR) - Beliefs about Medicines Questionnaire- specific (BMQ-specific) - Clinical Global Impression Bipolar Severity of Illness (CGI-BP-S) - Researcher-developed scale for Intention to use medications - Montgomery Asberg Depression Rating scale (MADRS) - Medication Adherence Rating Scale (MARS) - Perceived Behavioural Control (PBC) - Plasma level of mood stabilizer - Quality of Life in Bipolar Disorder Scale (QOL.BD) - Self-monitoring -Self-reported Behavioural Automaticity Index (SRBAI) - Young Mania Rating Scale (YMRS)		- Improvement in medication adherence - Improvement in all outcome measures in experimental group
Rezaei et al. 2018^78^	RCT	- Communication skills questionnaire - General Health Questionnaire (GHQ-28)	- Improvement in communication skills - Improvement in GHQ score	
Saberi et al. 2018^79^	Quasi-experimental	- Scale to Assess Unawareness In Mental Disorder (SUMD) - Young Mania Rating Scale (YMRS)		- Increase in insight

† RCT = Randomized controlled trial

## Limitation

The strength of this study is reviewing all interventions with psychoeducation as part of the package offered to patients and their families. Capturing all the core elements of psychoeducation intervention for patients suffering from SMD and their families is another strength of this study, which is useful in planning services. 

One limitation of this review is the lack of reported information on a number of variables, such as the educational level of caregivers, the relationship of caregivers to patients, the number of people who conducted the psychoeducational sessions and their professional capacity in several of the included studies. These variables are important in planning socially and culturally adaptable psychoeducation programs with limited resources. Another limitation is that the review did not include the research results of unpublished theses and dissertations topics in this area.

***Implications***

The main purpose of this review was to gather information on studies conducted in Iran to provide a roadmap for the implementation of psychoeducational programs for patients suffering from SMD and their families. This information can be used as an example for other LMICs.

Our review has a promising capacity in the area of patient and family psychoeducation in Iran. However, the main issue is still the implementation of such programs. Few pilot studies conducted in the newly developed community mental health centers in Iran show promising trends for the future (83). However, the important question that still remains is how many family and patient psychoeducation programs are part of ongoing routine clinical practice in Iran’s mental health system.

One of the barriers to feasibility in LMICs is the educational level of participants in psychoeducational intervention (7). In studies that provided information on the level of education, there is a percentage of participants with no or minimum literacy level (8 studies). A number of studies required at least a few years of education for the patient/family to be able to participate in the study (Table 1). In reality, that is not the case for all the patients or families. This is an important issue that should not prevent them from getting the help and support they need to cope with the illness.

Brief psychoeducational interventions in which patients and family members are provided with support and information about medication, the illness, and management strategies improve compliance, decrease relapse, and decrease readmission rates. This outcome is consistent in a number of studies included in the review as well as in other references (9).

To overcome difficulties in the implementation of psychoeducation interventions considering the limited resources, the incorporation of a level approach can be one useful way to involve patients and families. Initial contact, assessment, and general education built on the patient and families’ acceptability of services and the engagement process can decrease attrition rates (84). Discharge planning, as well as one session of psychoeducation during hospitalization are two examples of a leveled approach, which can facilitate further involvement with mental health services (33, 46).

There is a lack of information regarding training and supervision of mental health professionals while conducting psychoeducational sessions in most of the included studies. One study focused on service development with detailed information on training and supervision (76). Unfortunately, this is another important variable missing in the translation of program findings into practice in real-world settings.

Translating research findings into “real world” settings and improving the context of interventions plays a central role in the implementation process. To promote large-scale use and sustainability of an intervention, factors that describe various aspects of how the implementation of a program occurs and which important strategies facilitate the delivery are essential (85). Unfortunately, a number of included studies did not provide information on a number of key variables of psychoeducation which was part of their intervention. 

Based on studies included in this review, the majority of participants were male and the majority of the caregivers were female. Also, based on the results, low educational level should be considered in planning educational programs. Further, it was found that different methods of psychoeducation and mixed interventions are being used to provide psychoeducation to patients and their families. A number of possible contents are available in Farsi for psychoeducational sessions. In more than 40 studies, different mental health professionals were involved who could actively participate in capacity building and implementing psychoeducational intervention into routine practice in their workplace.

## Conclusion

This review included all studies that mentioned psychoeducation as part of their intervention. Although there are differences in the format and structure of education offered to families and their patients, the common factors of psychoeducation intervention provide a broad framework for future research as well as planning psychoeducation in community settings. To plan the implementation of family and patient psychoeducation, this review provides a basic structure including information extracted from studies on caregivers, interventions, manuals, and mental health personnel. This article has reviewed studies with a focus on the context and factors affecting implementation, such as the educational level of consumers and their families or the relationship of the main caregiver, which is important for the future planning of psychoeducational programs. 

Pragmatic and qualitative evaluations of appropriately adopted interventions that focus on feasibility and acceptance are necessary, given the promising outcome of studies published in Iran and other countries. Using information to guide the decision-making process for the service delivery of psychoeducation intervention for patients and their families is a priority for mental health services. In our opinion, based on evidence, even with limited resources, it is no longer acceptable to deprioritize some forms of psychoeducation for patients and their families in clinical settings. 

Future research with a focus on the implementation process and service development is much needed to facilitate the availability of psychoeducation to all patients suffering from SMD and their families in mental health settings in Iran.
